# Whole‐body heat stress and exercise stimulate the appearance of platelet microvesicles in plasma with limited influence of vascular shear stress

**DOI:** 10.14814/phy2.13496

**Published:** 2017-11-09

**Authors:** Eurico N. Wilhelm, José González‐Alonso, Scott T. Chiesa, Steven J. Trangmar, Kameljit K. Kalsi, Mark Rakobowchuk

**Affiliations:** ^1^ Centre for Human Performance, Exercise, and Rehabilitation College of Health and Life Sciences Brunel University London Uxbridge United Kingdom; ^2^ Division of Sport, Health and Exercise Sciences Department of Life Sciences Brunel University London Uxbridge United Kingdom; ^3^ Faculty of Science Department of Biological Sciences Thompson Rivers University Kamloops British Columbia Canada; ^4^ Present address: Departamento de Desportos Universidade Federal de Pelotas Pelotas Brazil; ^5^ Present address: Institute of Cardiovascular Science UCL London United Kingdom; ^6^ Present address: Department of Life Sciences University of Roehampton London United Kingdom

**Keywords:** Cycling, dynamic knee extensor exercise, microparticles, passive heating, shear stress

## Abstract

Intense, large muscle mass exercise increases circulating microvesicles, but our understanding of microvesicle dynamics and mechanisms inducing their release remains limited. However, increased vascular shear stress is generally thought to be involved. Here, we manipulated exercise‐independent and exercise‐dependent shear stress using systemic heat stress with localized single‐leg cooling (low shear) followed by single‐leg knee extensor exercise with the cooled or heated leg (Study 1, *n* = 8) and whole‐body passive heat stress followed by cycling (Study 2, *n* = 8). We quantified femoral artery shear rates (SRs) and arterial and venous platelet microvesicles (PMV–CD41^+^) and endothelial microvesicles (EMV–CD62E^+^). In Study 1, mild passive heat stress while one leg remained cooled did not affect [microvesicle] (*P* ≥ 0.05). Single‐leg knee extensor exercise increased active leg SRs by ~12‐fold and increased arterial and venous [PMVs] by two‐ to threefold, even in the nonexercising contralateral leg (*P* < 0.05). In Study 2, moderate whole‐body passive heat stress increased arterial [PMV] compared with baseline (mean±SE, from 19.9 ± 1.5 to 35.5 ± 5.4 PMV ^.^ *μ*L^−1.^10^3^, *P* < 0.05), and cycling with heat stress increased [PMV] further in the venous circulation (from 27.5 ± 2.2 at baseline to 57.5 ± 7.2 PMV ^.^ *μ*L^−1.^10^3^ during cycling with heat stress, *P* < 0.05), with a tendency for increased appearance of PMV across exercising limbs. Taken together, these findings demonstrate that whole‐body heat stress may increase arterial [PMV], and intense exercise engaging either large or small muscle mass promote PMV formation locally and systemically, with no influence upon [EMV]. Local shear stress, however, does not appear to be the major stimulus modulating PMV formation in healthy humans.

## Introduction

Microvesicles are small plasma membrane‐derived vesicles released by most cells and have been identified as biomarkers of parental cell phenotype (Jimenez et al. 2003). Microvesicle appearance in the circulation is a common response to several physiological disturbances (Sossdorf et al. 2011; Boyle et al. 2013; Jenkins et al. 2013; Augustine et al. 2014), but the complete relevance of this phenomenon is still being investigated. Although previously linked to pathophysiological conditions (Boulanger et al. 2001; Vanwijk et al. 2002), increased circulating concentrations of platelet‐derived microvesicles (PMVs) have also been observed during and after single bouts of exercise (Sossdorf et al. 2010, 2011; Chaar et al. 2011; Wilhelm et al. 2016), and it has been shown that PMVs can support revascularization and deliver growth factors to the vascular endothelium in vivo (Brill et al. 2005). Furthermore, microvesicles released during exercise seem to stimulate endothelial repair and angiogenesis in vitro (Wilhelm et al. 2016), but the mechanisms by which exercise stimulate the release of these microvesicles remain largely unknown.

Platelets express membrane glycoproteins (GP), such as GPIbα, which have been implicated in transducing external shear stress, and stimulation of platelets with shear forces promotes PMV formation and appearance ex vivo (Miyazaki et al. 1996; Reininger et al. 2006), suggesting that acute increases in vascular shear stress may be a major mechanism triggering PMV appearance. Because local intravascular shear forces increase dramatically during exercise (Tanaka et al. 2006; Padilla et al. 2011; Simmons et al. 2011), and a positive relationship between plasma vascular shear rate (SR) and [PMV] has been observed in resting upper limbs during cycling (Wilhelm et al. 2016), shear stress may be considered a putative stimulus leading to platelet vesiculation during exercise. On the other hand, a rise in plasmatic concentrations of endothelial‐derived microvesicles (EMVs) in response to exercise has been reported within some (Sossdorf et al. 2011; Kirk et al. 2013; Lansford et al. 2015) but not all conditions (Sossdorf et al. 2010, 2011; Chaar et al. 2011; Lansford et al. 2015; Wilhelm et al. 2016; Rakobowchuk et al. 2017), and is thought to reflect activation and damage in the face of an endothelial challenge (Jimenez et al. 2003; Jenkins et al. 2013). High shear forces have been reported to downregulate the release of EMVs in vitro through an endothelial nitric oxide synthase‐related pathway (Vion et al. 2013). If that is the case, exercise‐dependent and independent increases in vascular shear stress should stimulate the appearance of PMV, but not EMV, in vivo.

Intense large muscle mass exercise, however, induces systemic changes in circulating factors including catecholamines (Galbo et al. 1975; Rosenmeier et al. 2004; Tschuor et al. 2008), angiotensin II (Burger et al. 2011) and cytokines (Jimenez et al. 2003; Abid Hussein et al. 2008), which are known microvesicle production agonists in vitro and thus act as confounding factors when investigating whether shear stress is a major mechanism linked to PMV release during exercise. Fortunately, dynamic knee extensor exercise can be used to address this shortcoming since a small muscle mass exercise induces a relatively smaller systemic neuroendocrine and cytokine response compared with larger muscle mass exercise (Mourtzakis et al. 2004; Rosenmeier et al. 2004), while inducing marked local hemodynamic adjustments. For instance, intense single‐limb knee extensor exercise brings about only minor adjustments in circulating catecholamines and can increase thigh blood perfusion up to 7–8 L min^−1^, a relatively high value compared with ~10 L min^−1^ attained in the whole leg during maximal cycling (Mourtzakis et al. 2004; Mortensen et al. 2005, 2008; Calbet et al. 2007).

Vascular shear stress can also be modulated relatively independent of metabolism, albeit to a lesser degree, by changes in local tissue and core temperature. Passive heat stress imposes a significant cardiovascular challenge, with skin and limb tissue perfusion increasing as local and core temperature rises (Minson et al. 1998; Chiesa et al. 2015), which lead to elevated local vascular shear stress (Padilla et al. 2011; Simmons et al. 2011). Similar to small muscle mass exercise, there are only minor plasma catecholamine responses when passive heating is associated with localized single‐limb cooling (Chiesa et al. 2015). These unique features make passive heat stress and dynamic knee extensor exercise two pertinent approaches to investigate whether local elevation in vascular shear stress is a major mechanism linked to the increase in [PMV] observed with exercise.

There is in vitro evidence that shear forces stimulate PMV formation but in vivo cause and effect experiments, particularly in the context of exercise, are lacking. Considering the potential of circulating microvesicles as emerging biomarkers related to exercise, the aim of this study was to (1) investigate the dynamics of PMV and EMV appearance with heat stress and intense exercise across major arteries and veins and (2) to gain insight into whether shear stress is a potential mechanism leading to increased circulating PMVs with exercise. To this end, two experiments were performed in which participants were exposed to passive heat stress followed by a single‐limb knee extensor exercise with one leg kept cool throughout the main experiment or whole‐body passive heat stress that was then combined with intense large muscle mass exercise to modulate vascular shear stress responses. We hypothesized that increases in vascular shear stress would parallel changes in plasma PMV concentrations, so PMVs would increase from baseline during passive heat stress and exercise (Studies 1 and 2), and that this response would reflect local changes in vascular shear stress (Study 1). In contrast, the concentration of EMVs would remain similar to baseline as shear stress increased.

## Materials and Methods

Eight healthy physically active males (mean ± SD; 25 ± 4 years, 1.76 ± 0.04 m, 73 ± 5 kg – Study 1) and eight trained male cyclists (26 ± 6 years, 1.81 ± 6 m, 76 ± 9 kg – Study 2) were recruited to partake in two separate studies. The study design and aims were explained to each participant prior to obtaining informed written consent. The studies described in this report were approved by the Brunel University London Research Ethics Committee (RE4‐11 and RES4‐12).

### Study design

The first study was performed to examine the influence of local hemodynamic factors on microvesicle formation during passive heat stress and intense small muscle mass exercise, whereas the second study investigated the effect of passive heat stress and intense large muscle mass exercise on systemic PMV and EMV dynamics. Additional hemodynamic and temperature‐related data derived from these experiments have been published elsewhere (Chiesa et al. 2015; Trangmar et al. 2017).

In Study 1, eight healthy physically active males visited the laboratory on two separate occasions: (1) to familiarize themselves with a custom‐built single‐leg knee extensor exercise ergometer and determine their individual dynamic knee extensor exercise peak power output (PPO); and (2) to perform the main experimental visit during which the influence of heat stress and local exercise‐induced changes in SR on microvesicle dynamics were characterized. Maximal incremental knee extensor exercise tests with either the right or left legs were performed using a modified Monark ergometer. Briefly, PPO of the right and left quadriceps femoris muscles was determined during an incremental test consisting of 3‐min stages at 60 knee extensions per minute and 6 W increases at each stage. On the day of the main experimental visit, participants had their usual breakfast and arrived at the laboratory in the morning (between 7:00 am and 9:30 am). Participant's radial artery of the right wrist was cannulated (18 gage catheter, 16 cm, Multi‐Med M2716HE, Edwards Lifesciences), and intravenous cannulas were placed in the femoral vein of each leg in the retrograde direction under ultrasound guidance with the tip of the cannulas located approximately 1 to 2 cm distal to the inguinal ligament. After instrumentation and a period of rest in the supine position, participants were exposed to heat stress for 1 h by wearing a suit (perfused with hot water, 50°C) covering their torso and right leg, whereas the left leg remained cool through the application of ice packs (KoolPak, Warwickshire, UK). Participants drank water ad libitum to limit dehydration. Following passive heat stress, participants performed an incremental single‐leg knee extensor exercise protocol using the cooled leg, while the heated leg remained inactive. After 20 min of recovery, participants performed the same incremental knee extensor exercise protocol with the heated leg while the cooled limb remained inactive. Passive heat stress and left leg cooling were applied throughout the entire experimental trial, and blood samples were obtained simultaneously from the radial artery, cooled leg femoral vein, and heated leg femoral vein at baseline, after passive heating with single‐leg cooling, at the end of cooled leg exercise, recovery, and at the end of heated leg exercise.

In Study 2, eight trained cyclists (maximal oxygen uptake 4.5 ± 0.3 L min^−1^) attended the laboratory on two occasions: (1) to determine their aerobic PPO under control and heat stress conditions during incremental tests; and (2) for the main experimental visit, both of which involved upright cycling on an ergometer (Excalibur, Lode, Netherlands), as reported elsewhere (Trangmar et al. 2017). In brief, the incremental test was undertaken both in thermoneutral and heat stress conditions, with a 1‐h rest interval between protocols. During the main experimental trial, participants arrived at the laboratory after their usual breakfast and were instrumented. Cannulas (Logicath Quad Lumen, 18‐gage, MXA234X16X85; Smiths Medical International, UK) were placed into the brachial artery of the nondominant arm, and in the anterograde direction into the right common femoral vein using the Seldinger technique, with samples being obtained from a point proximal to the internal iliac vein. The study was conducted at a mild room temperature (~18–20°C) and humidity (~35–40%). Following a period of rest and baseline measurements, participants were exposed to passive whole‐body heat stress by circulating hot water through a perfused suit connected to a water circulator (Julabo F‐34, Seelbach, Germany, 50°C) until an increase of 1°C in body core temperature was achieved (~53 min of passive heat stress exposure). After passive heat stress, participants performed an incremental exercise to volitional exhaustion under heat stress while continuing to wear the water‐perfused suit, followed by recovery during which body temperature returned to baseline, and then a final incremental exercise protocol was completed under thermoneutral conditions (control exercise). The exercise protocol consisted of five 2.5‐min stages at percentages (20%, 40%, 60%, 80%, and 100%) of their condition‐specific PPO (i.e., 371 ± 33 and 321 ± 27 W for normothermic and heat stress PPO, respectively). Blood samples were obtained simultaneously from all cannulas at rest, after passive heating, and at 80% PPO of each exercise condition.

### Circulating microvesicle quantification

Microvesicles were measured in plasma. Citrated blood was initially centrifuged at 3000*g* for 10 min at 4°C to obtain platelet‐rich plasma, and platelet‐poor plasma was obtained for microvesicle quantification after a second centrifugation step at 15,000*g* for 10 min at 4°C. Samples were stored at −80°C until analysis. For analyses, samples were incubated at room temperature with a Fc receptor blocking solution for 10 min (Human TruStain FcX, BioLegend), followed by incubation with anti‐human CD41 (PE/Cy5) and CD62E (PE) monoclonal antibodies (BioLegend) for 30 min in the dark. After incubation, samples were washed with PBS and centrifuged at 17,960*g* and 4°C for 15 min. The microvesicle pellet was resuspended in buffer solution for imaging flow cytometric analysis using the ImageStream^X®^ Mark II (Amnis Corporation) at 60× magnification. Quantification of microvesicle concentrations was performed with IDEAS 6.1 software (Amnis Corporation) after single‐staining matrix compensation. Size calibration beads (Polysciences) were used to exclude nonmicrovesicle events greater than 1 *μ*m in diameter. PMVs were identified as CD41^+^ events with low SSC (Headland et al. 2014), whereas CD62E^+^ events were used to identify EMVs from activated endothelial cells, and a positive event threshold was established using single‐stained samples. Annexin‐V was not used as a generic marker of microvesicles since recent experiments demonstrate that only a subpopulation of microvesicles binds to annexin‐V (Connor et al. 2010). Previous experiments using a similar microvesicle quantification approach have demonstrated reliable measurements of PMV and EMV over time (Wilhelm et al. 2016).

### Blood variables

Heparinized blood was used to determine participants' hematocrit (Hct) and hemoglobin (Hb) concentration using a blood gas and metabolite analyzer (BL 800 FLEX, Radiometer, Denmark) according to the manufacturer's instructions. Changes in blood and plasma volume during the experimental protocols were calculated using equations described elsewhere (Dill and Costill 1974).

### Body temperatures

In the knee extensor study, intestinal temperature was measured with a wireless telemetry temperature sensor (HQInc, Palmetto, US) ingested 2–3 h prior the experimental trial. Thigh skin temperatures were obtained from each leg using wireless thermistors interfaced with data loggers (iButtons, Maxim). During the cycling study, core body temperature was estimated from the measurement of blood temperature in the common femoral vein using a thermistor connected to a thermocouple meter (TC‐2000, Sable Systems) and a data acquisition board (Powerlab, ADInstruments, Australia) and analyzed using LabChart software (version 8, ADInstruments, UK). Mean skin temperature was calculated as the weighted mean temperatures obtained from thermistors placed on the chest, arm, thigh, and calf (Ramanathan 1964), enabling the acquisition of systemic and leg skin temperatures.

### Local hemodynamics

Arterial diameters and mean blood velocities within the femoral artery were obtained by ultrasonography (Vivid 7, GE Logic, UK) using a linear array transducer for resting measurements during the cycling experiment and all measurements during the knee extensor study. Vascular SR was calculated as 4 × time‐averaged mean blood velocity/vessel diameter; and blood flow (BF) was the product of time‐averaged mean blood velocity × *π* × vessel radius^2^ × 60. Obtaining ultrasound measurements from exercising limbs required an extended acquisition time to ensure data quality, which limited our ability to record measurements from both limbs during knee extension protocols. As such, vascular SRs in the nonexercising limb during knee extensor exercise were estimated assuming that resting leg BF increased 75% during contralateral leg knee extensor exercise (Keller et al. 2003). The thermodilution technique was used to determine leg BF during exercise in the cycling study (Andersen and Saltin 1985; González‐Alonso et al. 2000). Because BF values obtained by ultrasonography and thermodilution techniques are highly correlated (*r* = 0.996) (Rådegran 1997), vascular SR could be estimated using the same equation stated above through approximations of blood velocities from thermodilution BF and assuming relatively unchanged femoral artery diameters, as reported in previous studies (Rådegran 1997).

### Statistical analysis

All descriptive data are presented as mean ± SEM unless otherwise stated. Repeated‐measures ANOVAs were performed to test differences within sampling sites and conditions. Post hoc tests were carried out whenever significant F‐ratios were observed for main condition effects, sampling site effects, or condition × sampling site interactions. The Dunnett's test for multiple comparisons was used to identify differences from baseline, and Bonferroni corrected t‐tests were performed to determine differences between sampling sites. Microvesicle arteriovenous (a‐v) differences were compared to an expected nought value with one sample t‐tests. To examine the relationship between circulating microvesicles and vascular SR, a within‐subject repeated‐measures multiple regression was performed as detailed elsewhere (Bland and Altman 1995), and results of individual analysis were illustrated as group averages for presentation purposes.

Methodological limitations mostly linked to issues with the thermodilution technique (for blood flow measurements) and in the acquisition blood samples (for MV quantification) reduced the number of complete datasets for analysis of selected variables. As such, the actual sample size entered for analysis is presented whenever missing data existed. Statistical analyses were performed using statistical software (SPSS version 20, IBM) with Dunnett's post hoc analyses calculated using GraphPad Prism (version 5.03, GraphPad Software). The significance level (*α*) for all tests was set at *α *< 0.05.

## Results

### Study 1: Responses to passive heat stress with single‐leg cooling, and intense knee extensor exercise with the cooled and heated leg

**Figure 1:**
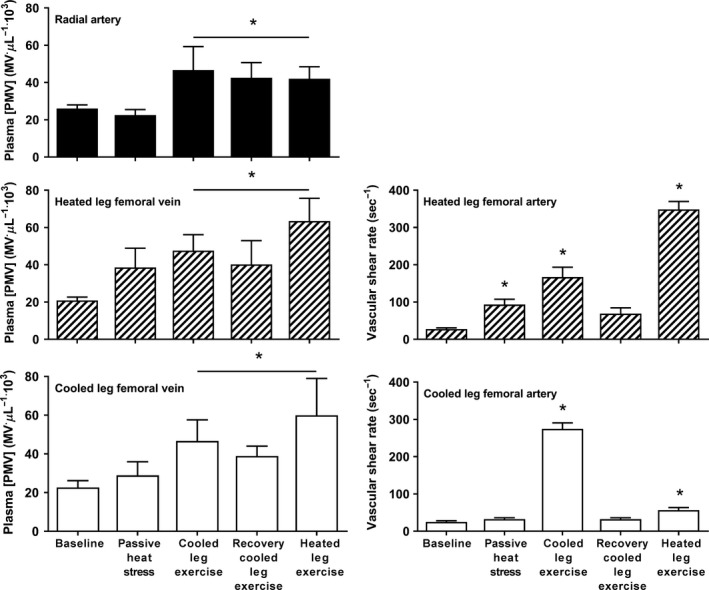
Effect of passive heat stress with single‐leg cooling and dynamic knee extensor exercise with the cooled and heated leg on arterial and venous plasma platelet microvesicle (PMV) concentrations and in femoral artery mean shear rate. [PMV] increased systemically during heat stress with cooled leg knee extensor exercise, and remained elevated thereafter in all sampling sites. Mean vascular shear rate and estimated shear rate (gray bars) increased throughout the protocol in the heated leg femoral artery, whereas with less pronounced changes in the cooled leg. Data are mean ± SEM for five participants. *Significant difference from baseline (*P* < 0.05).

One hour of passive heat stress with single‐leg cooling increased core temperature by 0.5°C compared with baseline, whereas skin temperature increased 9.7°C in the heated leg and 6.1°C at the systemic level, but dropped by 11.7°C in the cooled leg (*P* < 0.05, Table 1). Leg skin temperature compared with baseline remained elevated in the heated leg, and reduced in the cooled leg, during knee extensor exercise protocols (*P* < 0.05, Table 1). These thermal manipulations effectively produced distinct local hemodynamic adjustments, with femoral artery BF (Table 1) and SR (Fig. 1) being threefold higher than baseline during passive heat stress in the heated leg only (*P* < 0.05). Exercise with either the cooled or the heated leg elevated their vascular SRs by ~12‐fold (*P* < 0.05).

**Table 1:** Body temperature, blood flow and hematological data during passive heat with single‐leg cooling and dynamic knee extensor exercise

	Baseline	Passive heat stress	Cooled leg exercise	Recovery cooled leg exercise	Heated leg exercise
Core temperature (°C)	37.2 ± 0.1	37.7 ± 0.1*	37.8 ± 0.1*	37.9 ± 0.1*	38.0 ± 0.1*
Tsk (°C)					
Systemic	32.0 ± 0.2	38.1 ± 0.2*	38.3 ± 0.2	37.5 ± 0.1	37.6 ± 0.1
Heated leg	28.6 ± 0.9	38.3 ± 1.1*	–	36.3 ± 0.5*	36.3 ± 0.6*
Cooled leg	29.2 ± 0.3	17.5 ± 1.7*	23.8 ± 0.6*	–	–
LBF (L min^−1^)					
Heated leg	0.3 ± 0.02	0.9 ± 0.08*, †	1.5 ± 0.14*	0.7 ± 0.08*	3.7 ± 0.10*
Cooled leg	0.3 ± 0.02	0.3 ± 0.02	3.1 ± 0.15*	0.3 ± 0.02	0.5 ± 0.04
Blood volume change (%)					
Arterial	0	−3 ± 2	−8 ± 1	−7 ± 1	−9 ± 1
Venous (heated leg)	0	−3 ± 2	−10 ± 2	−6 ± 2	−8 ± 2
Venous (cooled leg)	0	−5 ± 2	−11 ± 4	−6 ± 2	−9 ± 2
Plasma volume change (%)					
Arterial	0	−8 ± 5	−15 ± 2	−17 ± 5	−22 ± 5
Venous (heated leg)	0	−6 ± 3	−16 ± 3	−14 ± 5	−16 ± 4
Venous (cooled leg)	0	−9 ± 3	−20 ± 3	−11 ± 3	−20 ± 5

Data are mean±SEM for 5–7 participants. Tsk, skin temperature; LBF, leg blood flow; heated LBF during cooled leg exercise, and cooled LBF during recovery and heated leg exercise are estimates; *P < 0.05 compared with baseline; ^†^ *P* < 0.05 compared with the cooled leg in the same condition.

**Figure 2:**
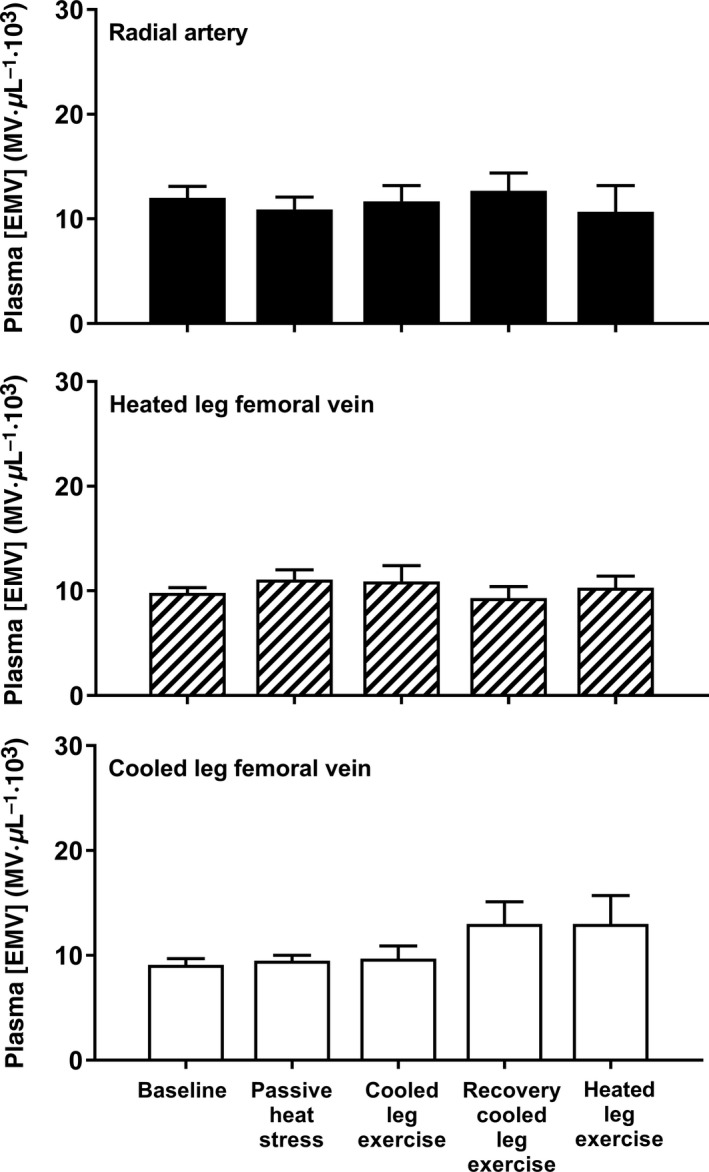
Effect of passive heat stress with single‐leg cooling and dynamic knee extensor exercise on arterial venous endothelial microvesicles (EMV) concentrations. Neither heat stress nor single‐leg knee extensor exercise affected the [EMV]. Data are mean ± SEM for five participants.

The concentration of PMVs was similar between arterial and venous samples at baseline, and passive heat stress with localized single‐leg cooling had no effect on plasma microvesicle concentrations. During knee extensor exercise, the [PMV] increased in the radial artery and in the venous circulation of both the exercising and nonexercising limbs by about twofold (*P* < 0.05, condition effect) (Fig. 1). This systemic increase in [PMV] was sustained above baseline during recovery and knee extensor exercise with the heated leg (*P* < 0.05). No changes took place in [EMV] at any sampling site (*P* ≥ 0.05, Fig. 2), and participants' microvesicle concentrations were similar in the arterial and venous samples throughout the experimental trial, resulting in no a‐v microvesicle difference (*P* ≥ 0.5).

### Study 2: Responses to whole‐body heat stress and intense cycling

**Figure 3:**
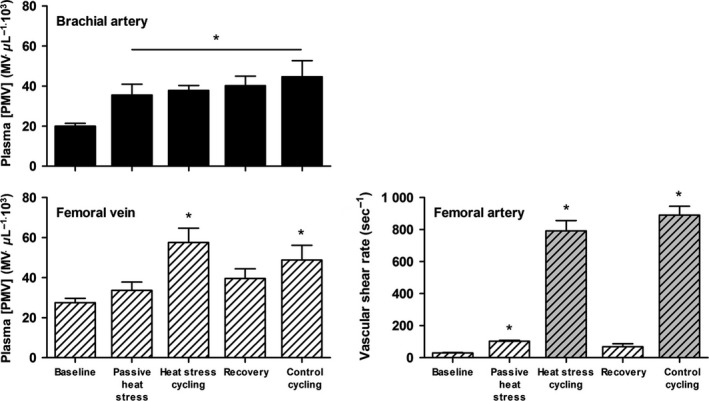
Effect of whole‐body heat stress and cycling under heat stress and thermoneutral conditions upon platelet microvesicle (PMV) and leg vascular shear rate. Arterial [PMV] increased with whole‐body heat stress and remained elevated during exercise, while venous PMVs only increased during cycling. Mean femoral artery shear rate was slightly elevated by passive heat stress, with large increases observed in estimates of vascular shear rate (gray bars) during exercise. Mean ± SEM for 6–7 participants. *Significant difference from baseline (*P* < 0.05).

Core body temperature increased >1°C with whole‐body passive heat stress and subsequent exercise (*P* < 0.05, Table 2). Estimated leg SR was elevated threefold with passive heat stress (*P* < 0.05, Fig. 3), increasing some 30‐ to 35‐fold from baseline during both heat stress exercise and thermoneutral control exercise (*P* < 0.05).

**Table 2:** Body temperature, two‐leg blood flow, and hematological responses to whole‐body passive heat stress cycling

	Baseline	Passive heat stress	Heat stress exercise	Recovery	Control exercise
Core temperature (°C)	36.5 ± 0.1	37.6 ± 0.1*	39.0 ± 0.1*	37.0 ± 0.1*	38.8 ± 0.1*
Tsk systemic (°C)	32.7 ± 0.4	38.7 ± 0.2*	36.7 ± 0.5*	32.3 ± 0.4	32.0 ± 0.5
Two‐LBF (L min^−1^)	0.6 ± 0.1	2.3 ± 0.2*	17.1 ± 1.3*	1.5 ± 0.4	19.2 ± 1.0*
Blood volume change (%)					
Arterial	0	−4 ± 1	−9 ± 1	4 ± 1	−6 ± 1
Venous	0	−4 ± 1	−10 ± 1	3 ± 1	−5 ± 1
Plasma volume change (%)					
Arterial	0	−6 ± 1	−16 ± 1	7 ± 1	−10 ± 2
Venous	0	−8 ± 1	−18 ± 1	6 ± 1	−10 ± 1

Mean±SEM for 6–7 participants. Tsk, skin temperature; LBF, leg blood flow; *P < 0.05 compared with baseline.

**Figure 4:**
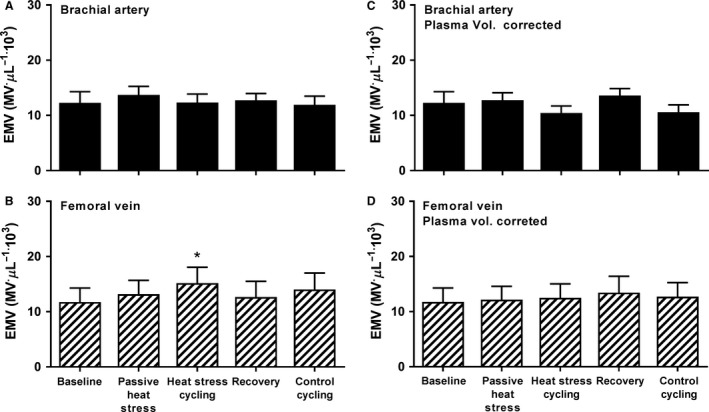
Effect of whole‐body heat stress and cycling on endothelial microvesicle (EMV) concentration sampled at the radial artery and femoral vein before (A and B) and after (C and D) correction for changes in plasma volume. No increase in venous EMV content was observed with whole‐body heat stress combined with large muscle mass exercise when changes in plasma volume were taken into consideration. Mean ± SEM for seven participants. *Significant difference from baseline (*P* < 0.05).

Baseline [PMVs] were higher in venous compared to arterial samples and whole‐body passive heat stress increased arterial [PMV] (*P* < 0.05, sampling site × condition interaction; Fig. 3), abolishing an initial a‐v PMV difference (*P* ≥ 0.05). Thereafter, arterial [PMV] remained elevated throughout the experimental trial (*P* < 0.05), and [PMV] sampled at the femoral vein increased dramatically during exercise with heat stress and in thermoneutral conditions (*P* < 0.05, Fig. 3), which caused an a‐v difference favoring PMV release from the active limbs during heat stress and exercise (*P* < 0.05), and a tendency toward release from exercising limbs during control exercise. No differences in [EMV] were observed with passive heat stress (*P* ≥ 0.05, Fig. 4), yet strenuous exercise combined with heat stress increased venous [EMV] (*P* < 0.05B). This increase, however, was abolished after correcting EMV values for changes in plasma volume (*P* ≥ 0.05, compared to baseline; Fig. 4D).

### Relationship between circulating microvesicles and shear rate

**Figure 5:**
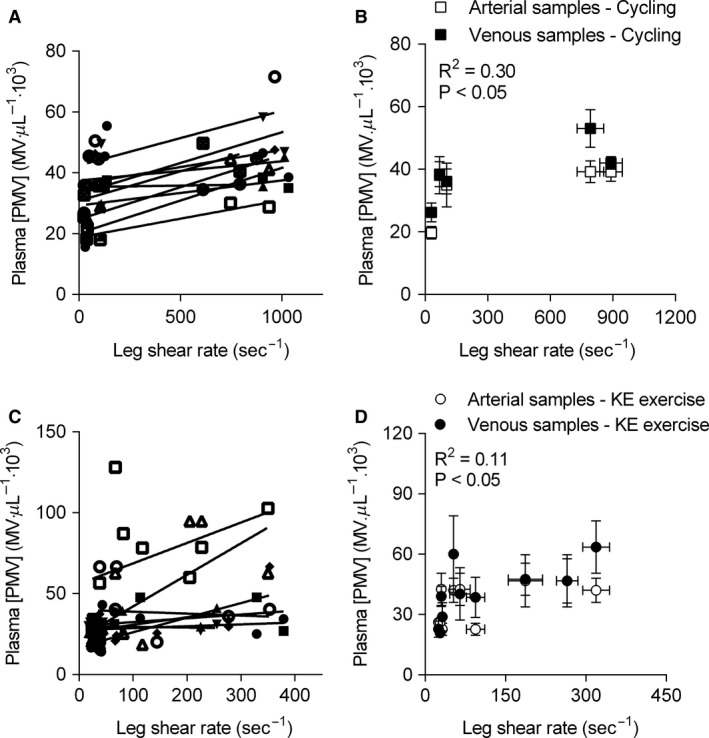
Relationship between platelet microvesicle (PMV) concentrations and femoral artery shear rate in the Cycling Study (A and B), and Knee Extensor Study (C and D). Vascular shear rate was estimated during passive heat stress and cycling exercise from individual within‐subject multiple regressions (A) or calculated during passive heat stress with simultaneous cooling of one leg and during single‐limb knee extensor (KE) exercise (C). Group averages (B and D) are displayed for illustration purposes. Compared with the cycling study, a reduction in the PMV explained variance is observed when data from active and contralateral inactive limbs are analyzed.

A within‐participant correlation between estimated SR, and arterial and venous [PMV] revealed that [PMVs] were moderately explained by vascular SR in the femoral artery during passive heat stress and exercise in the cycling study (*R* ^2^ = 0.30, *P* < 0.05, Fig. 5A and B). A weaker correlation between leg vascular SR and PMV was observed in knee extensor study, which included data from contralateral limbs during heat stress and exercise (*R* ^2^ = 0.11, *P* < 0.05, Fig. 5C and D).

## Discussion

We investigated the arterial and venous dynamics of PMVs and EMVs during passive heating and intense exercise, and we explored the relevance of vascular shear stress as a mediator of microvesicle release in healthy individuals. A consistent increase in both arterial and venous [PMV] during intense, small, and large muscle mass exercise was observed with a potential mismatch between local vascular shear rate and PMV appearance, suggesting that the formation of PMVs in exercising humans is not under direct regulation of local shear stress.

This study is the first to investigate the influence of heat stress upon PMV and EMV dynamics across human limbs. Mild levels of passive heat stress accompanied by single‐leg cooling (i.e., Study 1) had no impact on plasma microvesicle concentrations, but moderate whole‐body heat stress (Study 2) increased arterial [PMV] along with a tendency for its increase in venous samples, suggesting that platelets may release microvesicles into the circulation depending on the level of thermal strain. Passive heat stress has been used as an alternative cardiovascular therapy (Imamura et al. 2001; Brunt et al. 2016), with the capacity to evoke an increased anti‐atherogenic vascular shear stress profile (Chiesa et al. 2016) and to stimulate the mRNA expression of vascular endothelial growth factor and of other key proangiogenic mediators in human skeletal muscle (Kuhlenhoelter et al. 2016). Hence, one could hypothesize that microvesicles formed during whole‐body heat stress may resemble those produced during exercise, providing an adjunct effect leading to endothelial repair and adaptation with heat therapy. This hypothesis, however, warrants investigation.

The present findings are somewhat in agreement with animal models of heatstroke where increased annexin‐V^+^ MVs have been reported with passive heating (Bouchama et al. 2008). A recent publication, however, reported reductions in arterial [PMV] (CD62P^+^ – P‐selectin) in young men exposed to acute whole‐body passive heat stress, which elevated core temperature by +2°C (Bain et al. 2017). Currently, it is difficult to identify the reasons for such contradictory findings, but it may relate to the different sample preparation and storing protocols, distinct flow cytometer size resolution differences between studies, and the specificity of markers used for microvesicle population identification. For example, beyond being a valid platelet marker, P‐selectin is also expressed on the surface of activated endothelial, and although an exclusive platelet marker may be lacking when identifying microvesicles, one might speculate that the use of CD62P^+^ events as a gating strategy may not be as specific for PMV quantification as anti‐platelet glycoprotein markers used in this study and previous studies (Reininger et al. 2006; Sossdorf et al. 2011; Wilhelm et al. 2016).

Intense single‐leg knee extensor exercise promoted the appearance of PMVs at all vascular sampling sites by the end of cooled leg exercise, meaning that the [PMV] was elevated also in the arterial circulation and in the femoral vein of the contralateral nonexercising leg. The concentration of PMVs remained elevated above baseline throughout the protocol, indicating that exercise alters PMV dynamics by stimulating a sustained increase in microvesicles after exercise. These results agree with the cycling study presented here as well as past investigations where microvesicles remained elevated in peripheral blood during postexercise recovery (Sossdorf et al. 2010, 2011; Chaar et al. 2011; Wilhelm et al. 2016). They also support a systemic effect of exercise upon PMV appearance. The fact that plasma volume corrections did not abolish the increases in [PMV] substantiates these increases as not mere artifacts caused by hemoconcentration. Our results, therefore, advance the understanding of microvesicle dynamics by showing that intense exercise engaging either a small or large muscle mass augments circulating PMVs systemically.

During large muscle mass exercise, the increase in leg venous [PMV] was greater than that observed in the arterial circulation. As such, a negative a‐v PMV difference was observed during exercise under whole‐body heat stress, reflecting a net PMV release. This is a unique finding and suggests a rapid activation of platelets and microvesicle release as platelets travel through exercising limbs. This result, however, was not replicated in the knee extensor experiment, where no a‐v PMV differences were observed, and although one should consider the small sample size of knee extension study, these findings lead us to speculate that the amount of muscle mass engaged in exercise may influence the PMV dynamics. It is also worth noting that positioning of venous cannulas (anterograde vs. retrograde) differed between studies. Specifically, during the cycling experiment, additional regions that include the superficial tissues of the leg and lower abdominal/gluteal regions were sampled by the anterograde placement. It is currently unknown, however, if PMV turnover differs between these regions.

Changes in plasma [PMVs] may result from either an increased PMV release, a decreased PMV uptake, or a combination of both at the muscle level. Increased PMV release with exercise is most likely since mechanical forces (Miyazaki et al. 1996; Reininger et al. 2006) and biochemical agonists (Nomura et al. 2000; Tschuor et al. 2008) that stimulate the production of PMVs are known to increase during physical exertion. Evidence supporting microvesicle uptake also exists both in vitro and in vivo (Terrisse et al. 2010; Cantaluppi et al. 2012; Dasgupta et al. 2012); however, this has not been demonstrated during exercise. In animal models, PMVs appear to undergo rapid clearance (Rand et al. 2006) and might be internalized by endothelial cells in the pulmonary and systemic circulation (Terrisse et al. 2010; Dasgupta et al. 2012), but a reduction in PMV uptake seems less likely based on the tendency for a greater difference between venous and arterial [PMV] during exercise in our study.

The current findings also demonstrate that thermal stress coupled with large muscle mass exercise increased venous [EMV], whereas isolated quadriceps exercise did not induce any change. This result seems contradictory, since the release of microvesicles by endothelial cells is limited in situations involving high shear stress (Vion et al. 2013), and cycling produced almost twice as much estimated shear rate in exercising limbs compared with knee extensor exercise. Plasma volume corrections, however, abolished the observed [EMV] increase in the current experiment. This suggests the total number of EMVs circulating throughout the body within the plasma, and thus, their rate of release from endothelial cells did not change. Previous studies reporting increases in blood [EMV] in response to exercise have not described whether corrections for plasma volume shifts were performed (Sossdorf et al. 2011; Kirk et al. 2013; Lansford et al. 2015), and our findings, therefore, suggest that the eventual increase in plasma [EMV] does not necessarily represent endothelial activation and EMV shedding, but may result from hemoconcentration.

### Mechanistic insights

**Figure 6:**
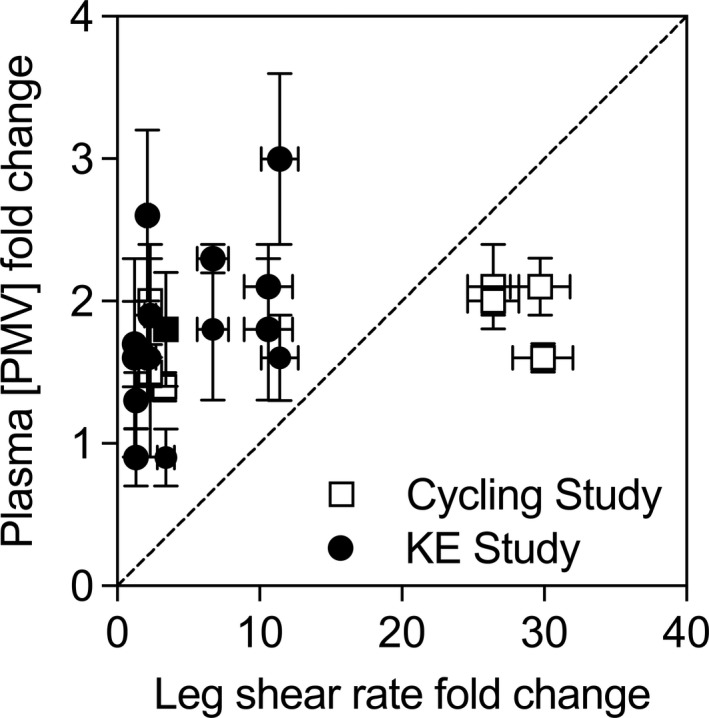
Representation of PMV and shear rate fold change in both experiments. The dotted line represents where the data should fall if there were a close relationship between shear rate and circulating PMV concentrations across a wide range (line slope 10:1).

Vascular shear stress seemed like a potential agonist stimulating PMV release during exercise, as platelets express mechanotransduction proteins and are stimulated to release microvesicles when exposed to increased shear forces ex vivo (Miyazaki et al. 1996; Reininger et al. 2006). As anticipated, intravascular shear stress was elevated in limbs exposed to passive heating, with local SR approaching similar values in the heated leg of both the knee extensor and the cycling study participants, whereas local leg cooling abolished the increase in SR during passive heat stress. Yet, an increase in circulating PMVs during passive heat stress was observed only when the level of core hyperthermia was moderate (+1°C), suggesting that the degree of heat stress may be important. Exercise stimulated the appearance of PMV both in exercising and inactive legs during the knee extensor study, but only markedly augmented SR in exercising limbs. This resulted in broad differences in SR, but relatively similar changes in circulating microvesicles between and within studies, as illustrated in Figure 6. Within‐subject correlations during heat stress and cycling revealed an association between [PMV] and vascular SR, which were similar to previous findings (Wilhelm et al. 2016). The explained variance, however, was reduced when data from inactive and exercising limbs (i.e., the knee extensor study) were incorporated into the model. The fact that the correlation is substantially lower in the single‐leg model, where confounding factors independent of shear stress are attenuated, further suggests that shear stress plays only a limited role in stimulating PMV release during exercise.

An increase in body temperature per se could be considered a mechanism inducing PMV appearance, as an elevation in procoagulant annexin‐V^+^ microvesicles has been observed in the circulation of baboons experiencing severe heat stress and heatstroke (rectal temperature ~44°C) (Bouchama et al. 2008), and moderate passive heat stress in humans (+1.3°C in core temperature) has been shown to increase procoagulant activity in the blood (Meyer et al. 2013). Experiments with ex vivo platelets, however, demonstrate that heat‐induced platelet hyperaggregability (an index of activation) requires incubation temperatures as high as 43°C (Gader et al. 1990), and it is unlikely that platelets of participants within the current studies were exposed to regional body temperatures higher than 40°C under passive conditions. Alternatively, adrenergic (Tschuor et al. 2008) and purinergic (Yegutkin et al. 2007) activation of platelets may stimulate PMV production during exercise, but these hypothetical mechanisms also need to be evaluated.

### Methodological considerations

It is worth noting that participants in Study 1 and 2 were not the same individuals, so caution must be taken when generalizing the present findings as it is uncertain whether microvesicle responses to heat stress differ between untrained and trained males. Furthermore, recent work by Bain and colleagues reported a reduction in arterial PMV and EMV concentrations of young males after exposure to passive heat stress eliciting a greater increase in body core temperature than in the present study (+2 vs. +1°C, respectively). Unfortunately, only pre‐ and postheat stress arterial blood samples were assessed and there was a lack of time‐control in that study, creating a rather unclear picture of the impact of heat stress on [PMV] in the circulation. Our current experiments assessed MVs across both venous and arterial vessels show no decline with heat stress, but a possible increase in PMV concentration in the circulation. To adequately determine the impact of heat stress upon circulating [PMV], future studies need to assess the PMV time course with passive heat stress to establish whether the PMV concentrations increase initially, and then decline with higher levels of hyperthermia as suggested by Bain et al. (2017).

## Conclusions

Although at first glance using whole‐body exercise the impact of shear stress upon platelet MV dynamics appears robust, this relationship becomes quite tenuous when examined using isolated limb exercise models. Furthermore, a weak relationship is supported by the experiments involving heat stress induced increases in shear stress, which show similar changes in [PMV] across diverse sampling sites that experience different shear rates. Finally, the observation of an increased [EMV] under the highest shear stress condition (whole‐body exercise accompanied by heat stress) is effectively removed when the influence of hemoconcentration is taken into account. Altogether, these observations suggest systemic release of PMVs with exercise and heat stress with minor influence of shear stress and raise the possibility of additional mechanisms that control their dynamics.

## Conflict of Interest

The authors declare no conflict of interests.
